# Acute Effects of Intratumor DNA Electrotransfer

**DOI:** 10.3390/pharmaceutics14102097

**Published:** 2022-09-30

**Authors:** Manya Bhandary, Amanda Sales Conniff, Kaitlyn Miranda, Loree C. Heller

**Affiliations:** Department of Medical Engineering, University of South Florida, Tampa, FL 33612, USA

**Keywords:** DNA electroporation or electrotransfer, molecular functions, cytoskeletal remodeling, inflammatory signaling

## Abstract

Intratumor therapeutic DNA electroporation or electrotransfer is in clinical trials in the United States and is under development in many other countries. Acute changes in endogenous gene expression in response to DNA or to pulse application may significantly modulate the therapeutic efficacy of the expressed proteins. Oligonucleotide arrays were used in this study to quantify changes in mRNA expression in B16-F10 mouse melanoma tumors four hours after DNA electrotransfer. The data were subjected to the DAVID v6.8 web server for functional annotation to reveal regulated genes and genetic pathways. Gene ontology analysis revealed several molecular functions related to cytoskeletal remodeling and inflammatory signaling. In B16-F10 cells, F-actin remodeling was confirmed by phalloidin staining in cells that received pulse application alone or in the presence of DNA. Chemokine secretion was confirmed in cells receiving DNA electrotransfer. These results indicate that pulse application alone or in the presence of DNA may modulate the therapeutic efficacy of therapeutic DNA electrotransfer.

## 1. Introduction

In vivo DNA electroporation or electrotransfer increases plasmid DNA (pDNA) delivery and expression in a wide variety of tissues types, including solid tumors [1]. The concept of intratumor therapeutic plasmid delivery in preclinical testing began with herpes simplex virus thymidine kinase gene and ganciclovir [2], but moved primarily into immune modulators [3]. Clinically, intratumor electrotransfer of plasmid DNA encoding IL-2 [4] and antiangiogenic metargidin peptide [5] has been tested. However, in both preclinical and clinical trials, interleukin 12 (IL-12) plasmid delivery has had great success therapeutically in many solid tumor types. Intratumor IL-12 plasmid electrotransfer is in Phase II clinical trials for metastatic melanoma alone or in combination with anti-PD-1 immunotherapy [6,7,8,9]. This combination therapy is also being tested with other solid tumor types such as Merkel cell carcinoma [10], triple negative and HER2+ breast cancers, and squamous cell head and neck cancer (clinicaltrials.gov (accessed on 2 September 2022)).

Non-coding control plasmid DNA can act as an immunotherapeutic adjuvant, increasing the expression of a variety of cytokines and chemokines. Control plasmid delivery induces independent antitumor effects, including tumor growth delay and complete regression as described in preclinical models of melanoma [11,12,13,14,15,16,17,18,19], lung carcinoma [20,21], fibrosarcoma [22], pancreatic carcinoma [23], breast cancer [24] and colorectal carcinoma [25,26,27,28]. This effect may be due to the activation of DNA-specific pattern recognition receptors (PRRs) following intracellular plasmid DNA detection. CpG motifs encoded in the plasmid DNA may bind and activate the endosomal PRR Toll-like receptor 9 (TLR9) [29]. Activation of germline encoded cytosolic or nuclear PRRs such as cyclic GMP–AMP synthase and an assortment of additional putative DNA-specific PRRs may induce the expression of proinflammatory cytokines and chemokines [30,31,32]. This expression may attract several types of immune cells to the tumor microenvironment.

The purpose of this study was to determine the acute global gene expression responses to intratumor electrotransfer of non-coding control pDNA in a syngeneic mouse model commonly used to simulate human melanoma [33]. We then confirmed the primary significant changes in vitro using B16-F10 mouse melanoma cells. This would enable a better understanding of the source of these antitumor effects.

## 2. Materials and Methods

### 2.1. Cell Line

B16-F10 mouse melanoma cells (CR6475, American Type Culture Collection, Manassas, VA, USA) were cultured in McCoy’s medium (Corning, Thermo Fisher Scientific, Waltham, MA, USA) and supplemented with 5% fetal bovine serum (FBS, Gibco, Waltham, MA, USA) in a 5% CO_2_ humidified incubator at 37 °C. The cells regularly tested negative for mycoplasma infection using the Myco-Sniff PCR Detection Kit (MP Biochemicals, Irvine, CA, USA).

### 2.2. Plasmid DNA (pDNA)

gWizBlank, an empty vector, was commercially prepared (Aldevron, Fargo, ND, USA) and diluted to a concentration of 2 mg/mL in physiological saline. Endotoxin levels were confirmed to be <100 EU/mg.

### 2.3. Mice and Intratumor Delivery

All procedures were approved by the University of South Florida Institutional Animal Care and Use Committee (protocol R2736, 2005). One million melanoma cells in 50 µL phosphate-buffered saline were injected subcutaneously in the left flank of female 7-to-8-week-old C57Bl/6J mice (Jackson Laboratories, Bar Harbor, ME, USA). Tumors were allowed to grow for eight days to a diameter of approximately 4 mm. Mice with tumors were randomized into groups then anesthetized using a mixture of 2.5% isoflurane and 97.5% O_2_. Four groups of four included mice with control untreated tumors, tumors injected with 50 µg plasmid DNA in 25 µL sterile physiological saline, tumors injected with 25 µL saline followed by pulse application, and tumors injected with plasmid DNA followed by pulse application. A 6-needle array controlled by an autoswitcher was fitted around the tumor, and delivery was immediately performed by the application of six 100 μs pulses with a voltage-to-distance ratio of 1300 V/cm and a frequency of 4 hertz using a legacy model ECM 830 Square Wave Electroporation System (BTX Harvard Apparatus, Holliston, MA, USA). Each mouse was monitored continuously until recovered from anesthesia, as indicated by their ability to maintain sternal recumbency and exhibit purposeful movement. Mice were humanely euthanized after four hours. Then, tumors were removed and snap frozen on dry ice.

### 2.4. Gene Expression Analysis

RNA was extracted from tumors disrupted in Trizol (Invitrogen, Carlsbad, CA, USA) using a rotor stator homogenizer then purified using RNeasy columns (Qiagen, Valencia, CA, USA). Quantified RNA from four tumors per group was pooled. Complementary DNA was generated using GeneChip IVT Express kits (Applied Biosystems, Waltham, MA, USA) and analyzed by a commercial service (SeqWright, Inc., Houston, TX, USA) using GeneChip Mouse Genome 430 2.0 arrays (Applied Biosystems).

### 2.5. Data Processing and Statistical Analysis

The data from the Expression Console software were processed per the supplied instructions (Affymetrix, Santa Clara, CA, USA). Briefly, every treatment group mas5-signal and mas5-detection was compared to the mas5-signal and mas5-detection from the control tumor samples. Signals < 100, as well as genes with AA (absent) mas5-detection in both treatment and control groups were disregarded. The fold change in the experimental groups was calculated by comparison with the control group, then sorted in descending order to allow for gene expression to be displayed. Downregulated genes were expressed by PA (present–absent, turn off) and PP (present–present, decrease), while upregulated genes were expressed by AP (absent-present, turn on) and PP (increase). The selection conditions of a significantly changed gene expression were based on a fold difference higher than absolute 2 and *p*-value after false discovery rate (FDR) correction <0.05. The differentially expressed genes (DEGs) with the highest fold change and q < 0.05 were analyzed using the DAVID (Database for Annotation, Visualization and Integrated Discovery) bioinformatics tool [34,35,36]. This database was used to perform the gene ontology (GO) functional annotation analysis, which classified the DEGs into molecular function. In the output given by DAVID, an FDR of < 0.05 was considered to be statistically significant. The *p*-values of selected GO terms were corrected using Benjamini–Hochberg correction described as adjusted *p*-values [37]. Relevant GO groups with adjusted *p*-values below 0.05 and N per group >5 were visualized using bar dot plot. Detailed analysis of genes belonging to selected ontological groups, with their expression Log fold changes (LogFC) are presented as chord plots using “GOplot” library packages and SRplot (http://www.bioinformatics.com.cn/, (accessed on 3 June 2022)) [38,39]. Gene symbols in the text are per the Mouse Genome Database [40]. Raw data and DAVID output files are available in Table S1.

### 2.6. Cytoskeletal Staining of B16-F10 Melanoma Cells

Cells were seeded into glass bottom 96-well plates and incubated for 4 h to allow attachment. The medium was replaced with 200 µL complete medium or medium containing 0.4 mg/mL pDNA. An electrode consisting of 2 parallel plates with a 2 mm gap (ACC-B15002, Leroy Biotech, Saint-Orens-de-Gameville, France) was inserted into the wells. Six 100 µs pulses at a voltage to distance ratio of 1300 V/cm and a frequency of 4 hertz were applied. After 30 min of incubation, cells were stained with 4′,6-diamidino-2-phenylindole (DAPI, Akoya Biosciences, Marlborough, MA) and Phalloidin iFluor 488 Reagent (Abcam, Cambridge, UK) per manufacturer’s instructions. Images were captured with a fluorescence microscope (BZ-X700E, Keyence Corp., Itasca, IL, USA). Fluorescence was quantified using the Hybrid cell count analysis application.

### 2.7. Chemokine Quantification by Bead Array

B16-F10 cells were suspended to a concentration of 2.0 × 10^7^/mL in 0.4 mg/mL pDNA. Six 100 μs pulses with a voltage-to-distance ratio of 1300 volts per centimeter and a frequency of 4 hertz were applied in cuvettes. Cells were incubated in complete culture medium for 4 h. One ml medium from 2 × 10^6^ cells was analyzed using a premixed multiplex panel (Mouse Cytokine/Chemokine Magnetic Luminex Assay, Millipore, Burlington, MA, USA) on a MAGPIX System (Luminex, Austin, TX, USA) per manufacturer’s instructions. All protein identifiers are per Uniprot [41].

### 2.8. Statistics

Statistical evaluation of the differences between groups and graph preparation was carried out using GraphPad Prism 9.1.0 (San Diego, CA, USA). Since the data were normally distributed, significance was determined by a one-way ANOVA test followed by a Tukey–Kramer post-test. A *p* < 0.05 was considered statistically significant. For the data obtained from microarray, differences were evaluated by statistical programs included in particular bioinformatic analyses.

## 3. Results

### 3.1. Tumor Electroporation Induces Gene Expression Changes Primarily Related to the Cytoskeleton

**Figure 1:**
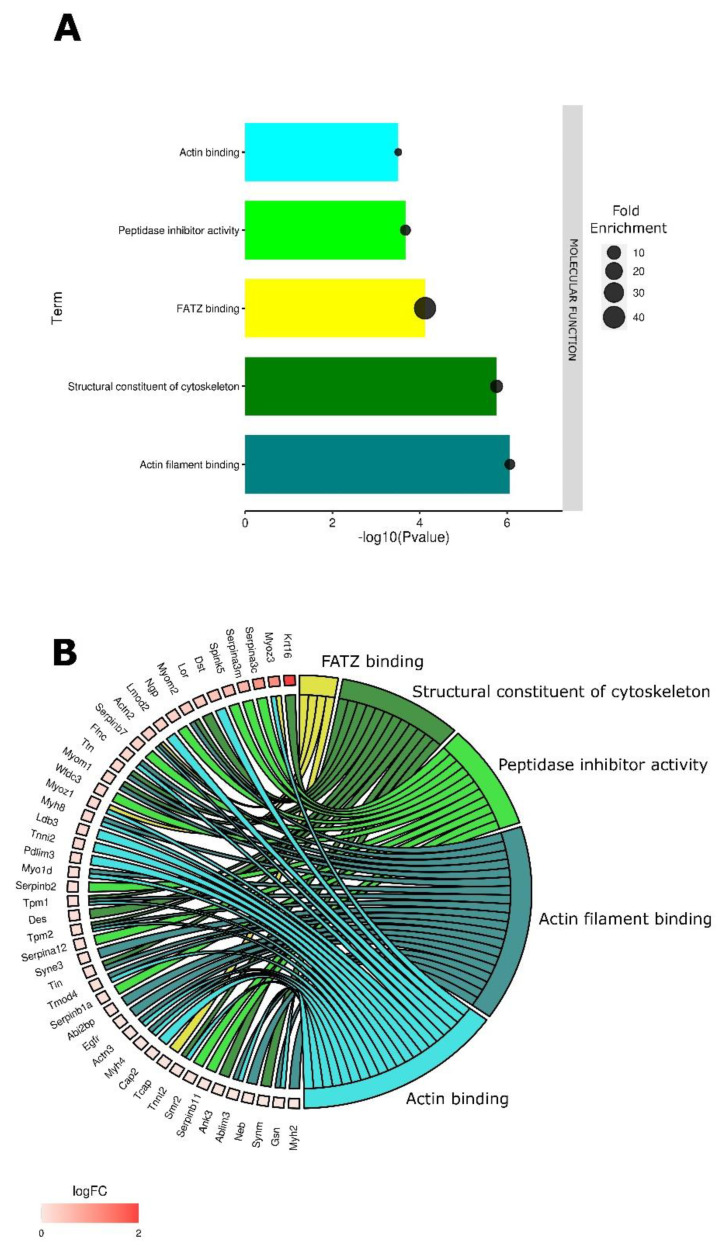
Enriched GO molecular function terms in electroporated tumors when compared to controls. (**A**) The bar dot plot shows the GO molecular function terms plotted in order of significance. (**B**) The chord plot shows the overlap of the DEGs contributing to these terms arranged in order of their fold change.

GO analysis indicated five molecular functions were significantly (*p* < 0.05) enriched four hours after pulse application alone. Four of the five functions related to cytoskeleton protein binding and structural integrity, including filamin-, actinin- and telethonin-binding protein of the Z-disc of skeletal muscle (FATZ) binding, structural constituent of cytoskeleton, actin filament binding, and actin binding (Figure 1A).

The DAVID Knowledgebase used in this study pulls from a variety of public databases [35,36]. Gene ontology terms are created using literature or data reviews or by automated methods [42] and are inherently incomplete and undergoing constant modification [43]. Related and overlapping DEGs may be represented in multiple GO terms. A chord plot shows the interrelation of specific DEGs (Figure 1B). Krt16 (keratin 6A), Myoz3 (myozenin 3), Serpina3c (serine peptidase inhibitor, clade A, member 3C), Serpina3m (serine peptidase inhibitor, clade A, member 3M), Spink5 (serine peptidase inhibitor, Kazal type 5) and Dst (dystonin) were upregulated more than 5-fold.

### 3.2. Cytoskeletal Changes

**Figure 2:**
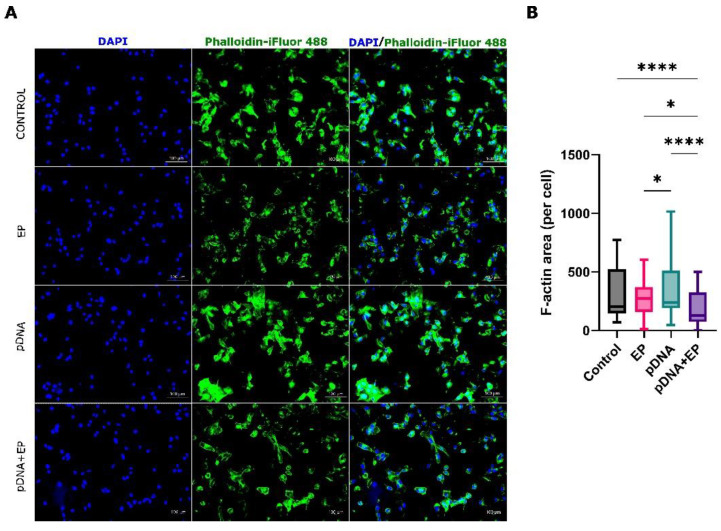
Cytoskeleton staining of B16-F10 cells pulsed in the absence or presence of plasmid DNA. Cells were pulsed in the presence of saline or pDNA (gWizBlank). (**A**) Phalloidin labeled F-actin (green) and DAPI nuclear staining (blue). Scale bar = 100 µm. (**B**) F-actin area per cell. EP, electroporation. *n* = 3 per group. **** *p* < 0.0001; * *p* < 0.05.

Given the significant regulation of gene expression in GO terms related to cytoskeletal molecular functions, we investigated cytoskeletal changes 30 min after delivery using fluorescent microscopy. Control B16-F10 cells showed their typical morphology with intact actin filaments (Figure 2A). Actin fibers were observed across the cells and over the nuclei as seen in the overlaid images. This cell morphology did not change with exposure to pDNA. Conversely, cytoskeleton changes were clearly observable in cells that were subjected electric pulses in the absence or presence of plasmid DNA. The actin fibers in both electroporation groups were visible mostly in the cell periphery and did not overlay the nuclei. Significantly reduced levels of actin per cell were detected in the electroporated groups (Figure 2B).

### 3.3. Plasmid DNA Injection of Tumor Induces Significant but Minimal Gene Expression Changes

**Figure 3:**
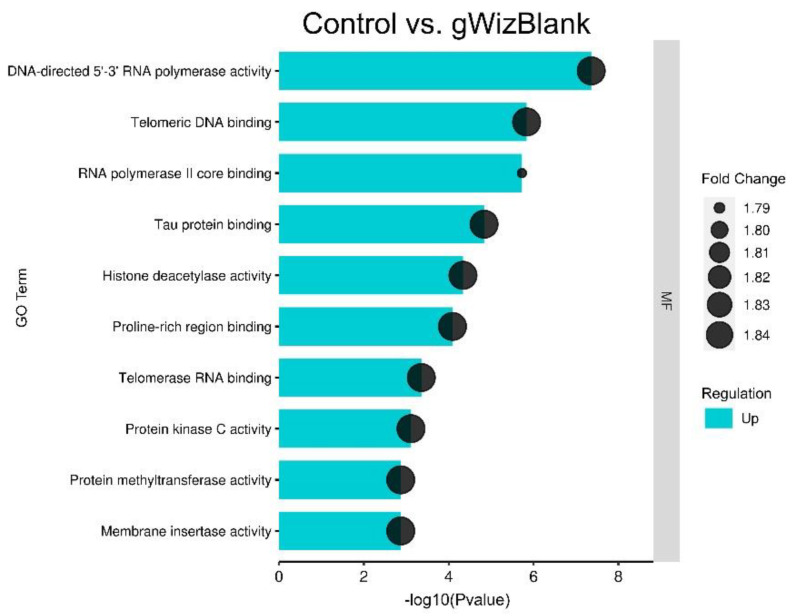
Enriched GO molecular function terms in tumors injected with pDNA when compared to controls. The plot shows the GO molecular function terms plotted in order of significance.

Figure 3 shows the ten most significant gene ontology terms regulated 4 h after pDNA injection. Several GO terms related to nucleic acid pathways were enriched, including DNA-directed 5′-3′ RNA polymerase activity, telomeric DNA binding, RNA polymerase II core binding, histone deacetylase activity, and telomerase RNA binding in order of significance. However, this regulation was modest (<2 fold).

### 3.4. pDNA Electrotransfer Is Associated with the Upregulation of Inflammatory Molecules

**Figure 4:**
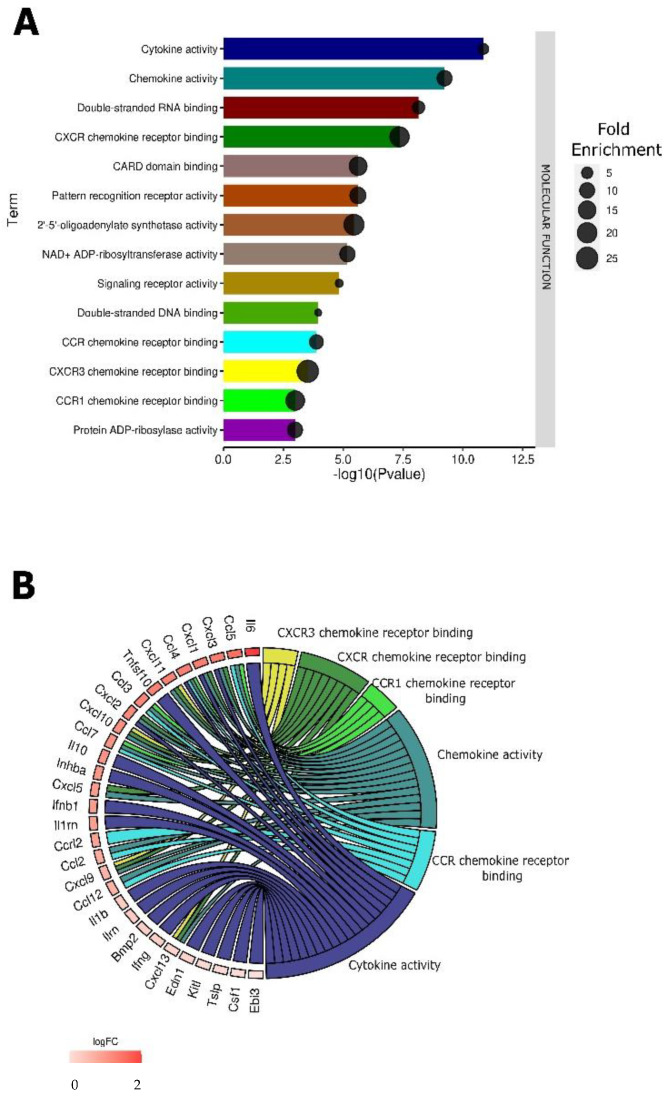
Enriched GO molecular function terms in tumors subjected to pDNA electrotransfer when compared to controls. (**A**) The bar dot plot shows the GO molecular function terms plotted in order of significance. (**B**) The chord plot shows the overlap of the DEGs contributing to chemokine terms arranged in order of their fold change.

As with pDNA injection (Figure 3), many terms related to nucleic acid binding and detection were regulated by pDNA electrotransfer (Figure 4A). However, the specific pathways differed. In order of significance, double stranded RNA binding, caspase recruitment (CARD) domain binding, PRR activity, 2′-5′- oligoadenylate synthetase activity, and double stranded DNA binding were regulated. However, in this group, several terms related to chemokine signaling were regulated, including C-X-C motif chemokine receptor (CXCR) binding, C-C motif chemokine receptor (CCR) binding, CXCR3 chemokine receptor binding, and CCR1 chemokine receptor binding (Figure 4B). To confirm these RNA expression data, we quantified chemokine secretion from B16-F10 cells 4 h after pDNA electrotransfer.

### 3.5. Validation of Chemokine Gene Regulation by Protein Production

**Figure 5:**
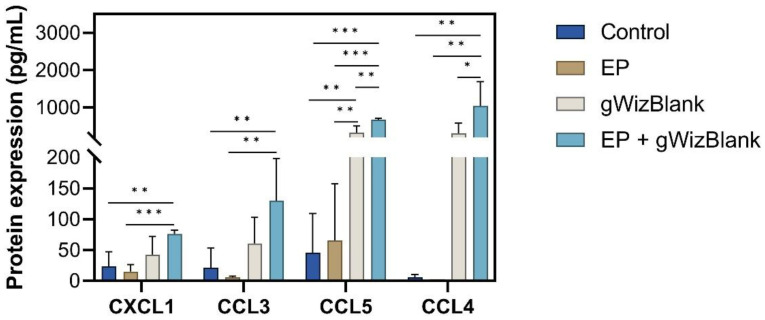
Detection of chemokine proteins in the medium of B16-F10 cells. EP, electrotransfer. *n* = 4–5 per group. *** *p* < 0.001; ** *p* < 0.01; * *p* < 0.05.

Fourteen chemokine or chemokine receptors mRNAs within this expression signature were upregulated more than 10-fold: Il6 (interleukin 6), Ccl5 (chemokine (C-C motif) ligand 5), Cxcl3, Cxcl1, Ccl4 (chemokine (C-C motif) ligand 4), Cxcl11, Tnfsf10 (tumor necrosis factor (ligand) superfamily, member 10), Ccl3 (chemokine (C-C motif) ligand 3), Cxcl2 (chemokine (C-X-C motif) ligand 2), Cxcl10 (chemokine (C-X-C motif) ligand 10), Ccl7 and Il10 (Figure 4B). We therefore quantified a subset of chemokine proteins secreted from B16-F10 cells 4 h after pDNA electrotransfer (Figure 5). Significant increases in CXCL1, CCL3, and CCL4 production were detected after pDNA electrotransfer. CCL5 was secreted after any DNA exposure. These results confirmed the observed mRNA regulation. Although RNA expression was increased, the CCL2, CXCL2, and CXCL10 proteins were not regulated at this time point.

## 4. Discussion

Microarray analysis was performed to evaluate the differences in gene expression between mouse melanoma tumors receiving electric pulses, backbone plasmid DNA injection, or pDNA electrotransfer when compared to control tumors. We found that two groups were highly regulated and enriched several gene ontology molecular functions that were statistically significant (*p* < 0.05). Pulse application alone dysregulated pathways related to cytoskeleton remodeling. We confirmed cytoskeletal changes microscopically in melanoma cells in both pulsed groups. Plasmid DNA electrotransfer induced dysregulation of a number of gene ontology terms related to immune signaling. Since several of these terms were related to chemokine signaling, we confirmed chemokine protein secretion by melanoma cells.

Four hours after pulse delivery, several molecular functions associated with cytoskeletal binding were regulated. This may consist of cell recovery to short-term pulse-driven actin rearrangements. Changes in cell morphology induced by several pulse types have been documented previously [44]. Using similar but less intense pulses to those used in this study, an early study demonstrated that cytoskeletal effects are observed within 5 min in endothelial cells. Actin staining is observed at the cell periphery, but the cells regained their original appearance within two hours [45]. Using pulses similar to those used in this study, the endothelial cell cytoskeleton transiently reorganized into fibers confined to the outer membrane within 2 h [46]. These cells recovered by 24 h after pulse delivery. Our observation that the cytoskeleton reorganized to the cell periphery after pulsing confirmed these observations in melanoma cells.

The application of other pulse types causes similar transient cytoskeletal effects. An early study in nanosecond pulse application demonstrated transient actin cytoskeletal rearrangement within one hour; the cell cytoskeleton recovered by 5 h [47]. These pulses induced intense cytoskeletal damage, as indicated by a speckled appearance [48]. Millisecond pulses induce actin changes in B16-F10 cells [49]. Transient cytoskeletal changes were observed in breast cancer cells after delivery of an 8 min constant current pulse [50]. The changes were reduced in fibroblasts, which demonstrates cell-type-specific aspects of this effect. The actin cytoskeleton is a major barrier to intracellular plasmid DNA movement [51]. Pulse-driven cytoskeletal rearrangements may participate in intracellular DNA movement [52].

In this study, we demonstrate that morphological changes in response to pulse application may not only occur in cells in vitro, they also may occur in the heterogenous environment of tumor tissue. Several groups have demonstrated that this effect is transient, and cells recover. In this study, we demonstrate that changes in gene expression may be necessary for this recovery. A large number of actin-binding proteins control cytoskeletal assembly and disassembly, which in turn controls processes such as intracellular transport, endocytosis and cell survival [53]. The mRNAs of several of these actin-binding proteins were upregulated in our study, implying that these proteins are necessary for cellular recovery.

We observed that simple plasmid injection caused statistically significant but minimal dysregulation of gene expression. Several of the regulated GO terms were related to nucleic acid binding. These diverse but related pathways may have been induced by the presence of plasmid DNA. The regulation of similar GO terms related to DNA binding was amplified by electrotransfer. Intercellular plasmid DNA is bound by several DNA-specific PRRs [54]. PRR activation has been implicated in B16-F10 cells [55], other types of cancer cells [56], B16-F10 tumors [18,19], and several cell types in normal skin [57] in the induction of inflammatory signaling.

Several GO terms related to chemokine signaling were highly regulated. Previously, we determined that tumor cells in culture will secrete a subset of chemokines, confirming the differential mRNA expression. In mouse melanoma tumors, non-coding control plasmid DNA electrotransfer induces the secretion of CCL3 after 4 and 24 h and CCL4 after 4 h [16]. This study confirms that, while many cells in the tumor microenvironment may secrete these chemokines, they are secreted by B16-F10 cells. We also previously detected mRNA upregulation and CXCL10 protein secretion by B16-F10 cells in several growth environments 4 h after pDNA electrotransfer [55]. The expression of some chemokine proteins did not reflect the mRNA regulation. Secretion was measured at a single time point; other chemokines may be secreted with different kinetic profiles. Although expression is transient, the chemokine-receptor axes activated by pDNA electrotransfer may attract a variety of both pro- and anti-tumorigenic immune cells into the tumor [58,59,60], altering the tumor microenvironment. For example, CCL3, CCL4 and CCL5 [61] can directly or indirectly attract immunosuppressive myeloid-derived suppressor cells [62] as well as beneficial dendritic cells and effector T cells [63].

The stimulated immunological changes can clearly modulate the efficacy of an immunotherapy, potentially requiring pharmacological control strategies. Potentially, the plasmid sequence can be modified to avoid these effects. Removal of CpG motifs, the TLR9 ligand, reduces but does not abolish inflammatory signaling [64,65]. This may be due to the binding and activation of ubiquitously expressed cytosolic PRRs. Their DNA ligands are incompletely described but are often related to structure rather than sequence. We have shown that putative PRRs IFI204, ZBP1 and DHX9 directly and durably bind pDNA within minutes of transfection [54]. These factors make plasmid modification to avoid immune activation completely a difficult objective.

This study was performed in a single experimental model. Gene expression analysis was performed on tumors in vivo, while the remaining analyses we performed on tumor cells in vitro. The responses described here may vary in different tumor and tissue types and in different mouse strains. Mouse and human immunology differs significantly [66]. Based on the universal expression of DNA-specific PRRs, we can predict that some response will occur. However, we may not be capable of predicting these responses precisely.

## 5. Conclusions

It is well established that changes in cytoskeletal arrangement are induced by the application of electric pulses in several cell types including tumor cells. We demonstrated that the primary regulated gene expression pathways associated with pulse delivery related to cytoskeleton protein binding and structural integrity. Therefore, regulation of these pathways may be integral to cell recovery. We also demonstrated that these gene expression changes occur in B16-F10 melanoma tumors, implying that previous in vitro observations predict changes in vivo.

PRRS are germline encoded and ubiquitous receptors that may bind plasmid DNA and induce the expression of pro-inflammatory cytokines and chemokines. This expression may have a profound effect on the tumor microenvironment. It is important to understand the positive or negative therapeutic impact of intrinsic DNA-directed responses to gene therapies.

## Data Availability

The data presented in this study are available on request from the corresponding author.

## Supplementary Materials

The following supporting information can be downloaded at: https://www.mdpi.com/article/10.3390/pharmaceutics14102097/s1, Table S1: Summary; Table S2: Control vs. pDNA+EP raw affymetrix probes data; Table S3: Control vs. pDNA+EP DAVID output Increase of expression Gene ontology Molecular functions; Table S4: Control vs. pDNA raw affymetrix probes data; Table S5: Control vs. pDNA DAVID output Increase of expression Gene ontology Molecular functions; Table S6: Control vs. EP raw affymetrix probes data; Table S7: Control vs. EP DAVID output Increase of expression Gene ontology Molecular functions.
